# Chinese and Western Musical Training Impacts the Circuit in Auditory and Reward Systems

**DOI:** 10.3389/fnins.2021.663015

**Published:** 2021-07-21

**Authors:** Sijia Guo, Ke Peng, Rui Ding, Junchen Zhou, Yan Liu, Yao He, Yuhong Liu, Ke Li, Pei Liu, Cheng Luo, Jing Lu, Dezhong Yao

**Affiliations:** ^1^The Clinical Hospital of Chengdu Brain Science Institute, MOE Key Laboratory for Neuroinformation, University of Electronic Science and Technology of China, Chengdu, China; ^2^Center for Information in Medicine, School of Life Sciences and Technology, University of Electronic Science and Technology of China, Chengdu, China; ^3^School of Music Education, Xinghai Conservatory of Music, Guangzhou, China; ^4^Department of Imaging, The 306th Hospital of the People’s Liberation Army, Beijing, China; ^5^Department of Music Education, China Conservatory of Music, Beijing, China

**Keywords:** musical training, expertise, musician, music styles, auditory system, reward system, fMRI

## Abstract

Previous studies have provided evidence about the brain plasticity effects of musical training, however, the issue of how expertise in music styles induced by Chinese or Western musical training affects neuroplasticity and reward responses has been less considered, especially for subjects of Chinese origin. In this work, 16 musicians who trained in the Western music style (Western-trained musicians) and 18 musicians who trained in the Chinese music style (Chinese-trained musicians) were recruited as the musician group for the experiment, while 15 non-musicians were recruited as the control group. Using a paradigm that consisted of listening to Chinese and Western music and measurements using functional magnetic resonance imaging (fMRI) technology, we found that Chinese-trained musicians activated the bilateral superior temporal gyrus (STG) when listening to music, while Western-trained musicians activated the left STG. In addition, under the condition of listening to music with Chinese style, Chinese-trained musicians have a stronger functional connection in the circuit of the auditory and reward system than Western-trained musicians. The finding is opposite under the condition of listening to music with Western style. Interestingly, it seems that the circuit of Chinese-trained musicians is partial to the right STG, while Western-trained musicians show the opposite, i.e., a tendency toward the left STG. The influence of different music styles on experienced musicians is reflected by the functional activities and connections between the auditory system and the reward system. This outcome indicates that training in Chinese music style or Western music style affects the strategies of musicians when listening to music. Musical characteristics such as rhythm, melody and cultural attributes play an important role in this process. These findings, which provide evidence for functional neuroplasticity based on musical training, can enrich our insights into the musical brain.

## Introduction

Brain plasticity under the influence of music has been a focus of scholars due to its great potential influence on shaping our cognition. Musical training during childhood and adolescence can affect neural development and enhance abilities of speech-in-noise processing, language skills, etc (Rüsseler et al., 2001; Tervaniemi et al., 2001; Strait et al., 2012; Tierney et al., 2015; Lu et al., 2017). The benefits of musical training have effects across lifespans (Strait and Kraus, 2014). Musical training experience affects neural development (Elbert et al., 1995; Lu et al., 2017; Li et al., 2018), with influencing factors that include the age at which training begins (Ohnishi et al., 2001) and the instruments musicians study (Hund-Georgiadis and von Cramon, 1999; Pantev et al., 2001). Scientists have found that experienced musicians exhibit enhanced auditory cortical representations for musical training associated with their principal instrument (Pantev et al., 2001). Therefore, different instrumental trainings have different influences on musical processing, which influence our cognition and perception of musical components (Drake and El Heni, 2003; Bouwer et al., 2018). In addition, another reason for the difference in neural plasticity between musicians is that musicians listen to music with different styles when they are training different musical instruments. For example, Chinese music style and Western music style; the musical rhythms of Western classical music are considered highly regular and predictable (Levitin et al., 2012). Specifically, majority of Western music has regular beat levels, which include even time divisions and time intervals composed of simple ratios of 1:1 and 2:1 (Stevens, 2012). Beat is a fundamental feature of Western music and that the prominent characteristic of Western music is the regular subdivision of the beat. Typical Western music’s primary beat level has one or two levels of subdivision (London, 2012). Different from Western music style, music from Asian regions has irregular beat attributes (Stevens, 2012). The duration of one beat of Chinese music is sometimes based on one monosyllabic Chinese character. Under this circumstance, the instrumental performance of Chinese music usually corresponds to an irregular stress duration (Cao and Li, 2017). Chinese music style pays more attention to melody compared with Western music style. The application of melodic techniques such as atonality, microtones, pantonality and polytonality can be seen in its composition (Jiang, 1991).

Study indicates that music is culturally universal and culture-specific (Morrison and Demorest, 2009). Exposure to the particular melodic, harmonic, rhythmic, or timbral features of the surrounding musical culture leads to the enhancement of musical abilities, similar to children learning language (Morrison et al., 2003). People who have received musical instrument training since childhood can further enhance their musical abilities corresponding to the characteristics (rhythm, melody, etc.) of music style that they are exposed to and practice during daily training. Experienced musicians who have received Chinese or Western musical instrument training can distinguish the difference between Chinese and Western music styles based on their professional knowledge and abilities. Therefore, we suggest that both Chinese-trained musicians and Western-trained musicians have a greater understanding of the musical styles they are exposed to during their daily training than non-musicians and musicians who are trained in other types of music.

Previous neurological and pharmacological studies have shown that the reward system activates when subjects listen to music, and dopamine is the physiological basis of the reward response (Salimpoor et al., 2011; Raghanti et al., 2016; Ferreri et al., 2019). This process is related to the pleasure experienced when listening to music. Research has indicated that expectations can be generated by veridical (characteristic-specific) knowledge about a familiar genre. The predictability of musical characteristics possibly contributes to our enjoyment of music (Levitin et al., 2012). Furthermore, the reward responses are different when subjects are familiar or unfamiliar with the music (Freitas et al., 2018). Thus, the understanding of musical styles probably relates to the pleasure experienced in reward processing and the relatively dominant music characteristics in the music style may have a greater contribution in this process. The brain regions involved in this process include midbrain, abdominal and back lateral striatum, amygdala, prefrontal lobe, cingulate gyrus, etc. (Pantev et al., 1998; Koelsch et al., 1999; Blood and Zatorre, 2001).

Researchers have explored the difference in the neural activation among subjects from different cultures when they encounter cultural familiar/unfamiliar music. For example, Western musicians mainly activated bilateral motor regions in the culturally familiar condition and activated a right lateralized network of angular gyrus and the middle frontal gyrus in the culturally unfamiliar condition (Nan et al., 2006). However, musical interaction related to culture affects human responses to music during infancy (Morrison and Demorest, 2009). In this period, musical abilities are related to attention, memory, and the acquisition of musical information develop (Trehub, 2001). In addition, language learning has a transfer effect to music learning, and native speakers of different languages have different musical abilities (Zhang et al., 2020). Thus, recruiting subjects from different cultures may be disadvantageous to our study of the influence of training in Chinese and Western music styles on neural plasticity. At present, majority of the research on neural plasticity under musical training has been conducted on subjects with Western cultural backgrounds. It is necessary to study neural plasticity under musical training which conducted on subjects coming from other musical traditions as well. Therefore, the current study places its focus on musicians of Chinese origin, who were trained in either Western or Chinese music style.

In summary, we assume that different musical styles which musicians are exposed to during their daily training, have different influences on brain plasticity. Perhaps music styles affect the strategies of musicians when they listen to music. Additionally, in the process of listening to music with different styles, their brain areas related to the processing of musical characteristics have different connections with the reward system. Herein, we plan to recruit Chinese subjects to study the neural plasticity of musicians exposed to the corresponding musical styles due to long-term musical training.

## Materials and Methods

### Participants

**FIGURE 1:**
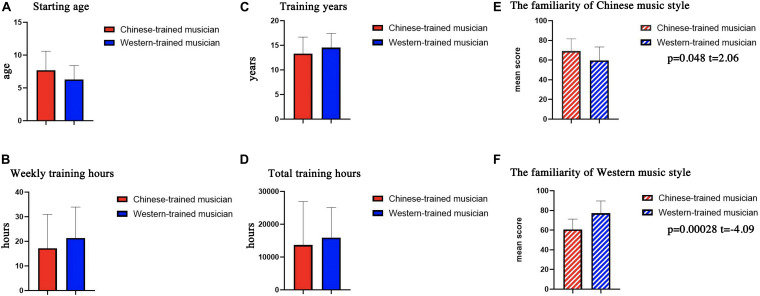
The comparisons of background information between Chinese-trained musicians and Western-trained musicians. **(A)** Starting age, i.e., the mean age at which participants started to learn how to play the instrument. **(B)** Weekly training hours, i.e., the mean hours they spend practicing every week reported by the participants. **(C)** Training years, i.e., the mean total number of years since they started to learn the instrument. **(D)** Total training hours, which is calculated by weekly training hours × the number of weeks in a year × training years. There was no significant difference between the two types of musicians in these results. **(E,F)** Musicians have significant differences in familiarity with Chinese and Western music styles. Specifically, compared with Western-trained musicians, Chinese-trained musicians are more familiar with Chinese music style. In addition, compared with Chinese-trained musicians, Western-trained musicians are more familiar with Western music style.

In this experiment, 49 subjects of Chinese origin were recruited among undergraduate and Master’s degree candidates at different universities. Background information, such as starting age, training years, weekly training hours, etc., were obtained using the Montreal Music History Questionnaire (MMHQ) (Coffey et al., 2011). Eighteen musicians who played the guqin or erhu were selected as the Chinese-trained musician group; sixteen musicians who played the piano were selected as the Western-trained musician group. Fifteen non-musicians who had no formal musical education and had never played a musical instrument were selected as the control group. All of the musicians were from the Chinese Conservatory of Music or the Central Conservatory of Music. The non-musicians were recruited from other universities. The age of initial musical training was approximately 7 years among the musicians. They trained several hours every day and did not study other instruments during their daily non-training hours. Information about the participants is provided in Table 1. The comparisons of background information between Chinese-trained musicians and Western-trained musicians are provided in Figure 1.

**TABLE 1:** Characteristics of the participants.

**Participants**	**Age (mean ± SD)**	**Age of musical acquisition (mean ± SD)**	**Musical training hours per week (h)**	**Cultural background**
Non-musician group	21.3 ± 2.0	No musical learning experience	Not applicable	Chinese
Western-trained musician group	20.3 ± 4.1	7.7 ± 2.9	21.4	Chinese
Chinese-trained musician group	20.5 ± 3.1	6.3 ± 2.1	17.2	Chinese

All of the subjects were right-handed according to the Edinburgh Handedness Inventory (Oldfield, 1971) and had normal hearing abilities. They were paid for participating in the study. The study was performed with the approval of the Ethics Committee of the School of Life Sciences and Technology at the University of Electronic Science and Technology of China. All procedures were conducted in accordance with approved guidelines. Before the experiment, all of the subjects were fully informed about the nature and procedures of the study, and informed written consent was obtained from participants prior to enrollment in the study.

### Experimental Tasks

**FIGURE 2:**
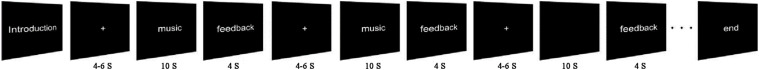
Experimental task. In each trial, the musical stimuli (10 pieces from Chinese musical pieces and 10 from Western musical pieces) and the control stimuli (10-s blank period) were presented in a pseudorandom order after presentation of a fixation cross for 4–6 s. The participants were instructed to provide feedback on their level of familiarity with music styles (familiar, ordinary, and unfamiliar) by using a keyboard within 4 s.

The musical stimuli in the experiment consisted of 20 pieces, each of which was 10 s in length. Considering the daily training courses of Chinese-trained and Western-trained musicians, ten pieces of Chinese music style were excerpted from traditional Chinese music (Ai Ying, Da Mo Shu Huai, etc.), and ten pieces of Western music style were excerpted from the Baroque period to the 20th century (Concerto Grosso in D Major, Memories of Constantinople, etc.). The musical stimuli were downloaded from the International Music Score Library Project^1^ and Netease Cloud Music^2^. For each musical stimulus, a 10-s excerpt that represented the most characteristic and recognizable segment of the music was selected. The musical features of the selected musical stimuli remained intact and true to the original version. The loudness of the musical pieces was fixed and comfortable for the subjects. All of the subjects had never listened to the musical stimuli used in the experiment before. The degree of familiarity with music mentioned later refers to whether the subjects are familiar with the style of the music they listen to. To eliminate irrelevant variables, purely instrumental music, e.g., that which did not contain vocal sections, was used during the entire experiment. In addition to the musical stimuli, a 10-s blank period was used as a control condition during which a blank screen without any auditory stimulation was presented. The subjects who participated in the experiment did not know these details in advance, ensuring they would not have a deliberated response to the music. Before the experiment, the subjects were instructed to avoid strong head movements and to carefully listen to the presented music. One practice trial was presented on the screen to confirm that the participants understood the task instructions (Figure 2). Each participant completed 10 trials of Chinese music, 10 trials of Western music and 5 trials of the control condition. Each trial consisted of presentation of a fixation cross (of 4–6 s; jitter was used to cause the brain-blood oxygen signal to return to baseline and to separate the stimulation time), 10 s for the stimuli to appear randomly and 4 s for the subjects to select one of the three response possibilities indicating different stages of familiarity with the musical styles (familiar, ordinary, and unfamiliar). Responses were accomplished by pressing a button on an MRI-compatible response box (if subjects could not distinguish the familiarity of the musical styles, they were instructed to respond with “ordinary”). The performance of the task was judged according to the responses of the subjects. The total duration of the task for each subject was 450–500 s. The tasks were designed and presented using the E-prime software, version 2.0 (Psychology Software Tools, Inc., United States) (Figure 2).

### fMRI Scans

Functional MRI scans were acquired using a 3T magnetic resonance imaging (MRI) scanner (Siemens MAGNETOM Trio 3T, Germany) with a standard GE whole head coil performed at the MRI Research Center of the 306th Hospital of the People’s Liberation Army. During scanning, foam padding and earplugs were used to reduce head motion and scanning noise, respectively. The functional images were acquired using gradient echo-planar imaging (EPI) sequences [slices = 30, averages/measurements = 1/244, echo time (TE) = 30 msec, repetition time (TR) = 2,000 msec, flip angle (FA) = 90°, field of view [FOV] = 210 mm × 210 mm, matrix = 64 × 64, acquisition voxel size = 3.3 mm × 3.3 mm × 4.0 mm, reconstructed voxel size = 3 mm × 3 mm × 3 mm, multislice mode/series: interleaved/descending, bandwidth = 2,232, and slice thickness/gap = 4 mm/0.8 mm] with an eight channel phased array head coil. To ensure steady-state longitudinal magnetization, the first five volumes were discarded. Subsequently, high-resolution T1-weighted images were acquired using a 3-dimensional fast spoiled gradient echo (T1-3D FSPGR) sequence (TR = 2,300 msec, TE = 2.98 msec, FA = 9°, matrix = 256 × 256, FOV = 240 mm × 256 mm, slice thickness/gap = 1 mm/0.5 mm, and slices = 176).

### Functional Imaging Analysis

#### Processing

The preprocessing and statistical analysis were performed using SPM8 software (Statistical Parametric Mapping)^3^. We conducted slice time correction, 3D motion detection and correction, spatial normalization to the Montreal Neurological Institute (MNI) template supplied by SPM, and spatial smoothing using an isotropic Gaussian kernel (8 mm full width at half maximum). To avoid MRI machine field effects and to eliminate the head movements of the participants, a series of preprocessing steps, including discarding the first five volumes, normalizing the images with an echo planar imaging template to the MNI atlas space (Evans et al., 1993), and resampling to 3 mm × 3 mm × 3 mm, were performed. Temporal bandpass filtering (pass band 0.01–0.08 Hz) was conducted using a phase-insensitive filter, which was used to reduce the effects of low-frequency drift and high-frequency noise. The time series was further corrected for the effect of six head motion parameters obtained in the realigning step.

#### Statistical Testing

**FIGURE 3:**
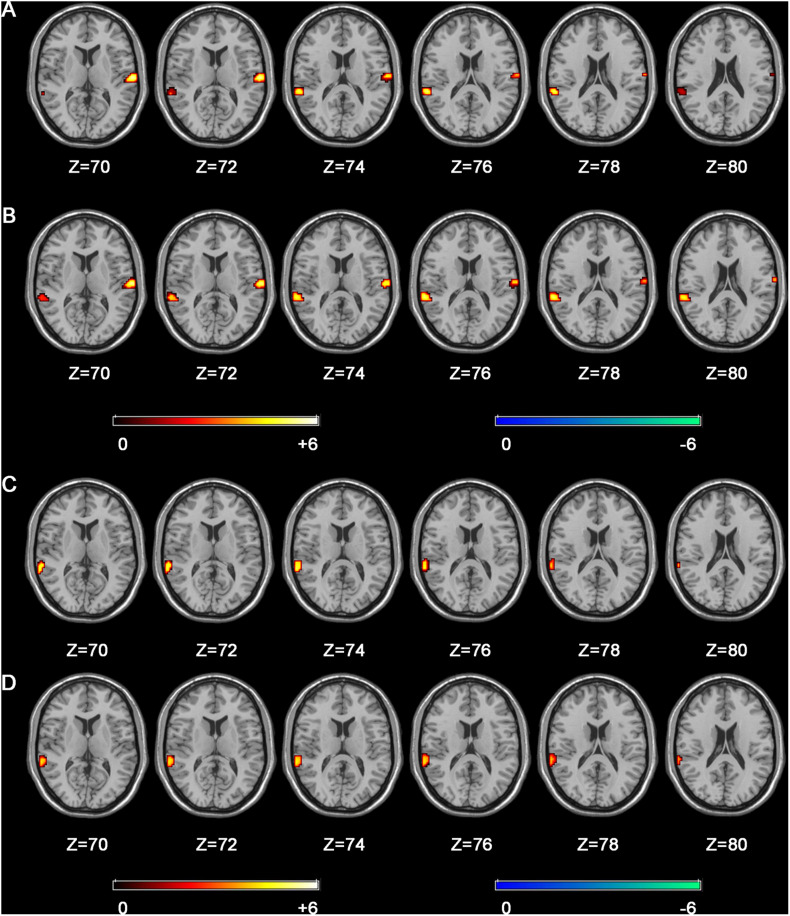
Comparison of brain activity during the experimental task when Chinese and Western-trained musicians listened to music with familiar and unfamiliar styles compared to non-musicians. Compared to non-musicians, Chinese-trained musicians have greater activation of the bilateral superior temporal gyrus, and Western-trained musicians have greater activation of the left superior temporal gyrus. **(A)** Chinese-trained musicians – music with Chinese style. **(B)** Chinese-trained musicians – music with Western style. **(C)** Western-trained musicians – music with Chinese style. **(D)** Western-trained musicians – music with Western style.

We compared the background information differences between Chinese-trained musicians and Western-trained musicians, including starting age, training years, weekly training hours, total training hours and familiarity with Chinese and Western music styles (Figure 1). For fMRI data, the second-level analysis embedded in SPM8 software was used. For the first level analyses, data were analyzed using a generalized linear model (GLM) for the three conditions (Western music style, Chinese music style and the control condition) for each subject. These conditions were modeled by a function convolved with the hemodynamic response function, which was used as the regressor to analyze the task. The six head motion parameters were included in the design matrix to control for movement-related artifacts. For the second level analyses, the contrast images resulted in the first level analyses were entered into group analyses for each of the corresponding contrasts. To further evaluate the differences of listening to music with Chinese or Western styles, comparisons were then made between Western-trained musicians vs. non-musicians and between Chinese-trained musicians vs. non-musicians under the conditions of listening to music with Chinese or Western style in order to ensure basic task-related activation by using a two-sample *t*-test (*p* < 0.05, false discovery rate (FDR)-corrected, and cluster size ≥621 mm^3^). The MNI space displayed the coordinates of the voxel with the maximal *T*-value corrected for the cluster size. Clusters whose values were below 23 were rejected. After analyzing the data, areas that were more activated during the experimental task were identified (Figure 3).

**FIGURE 4:**
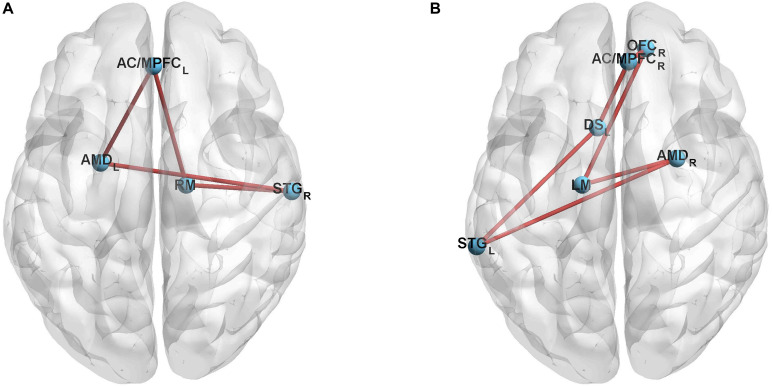
Comparison of the connections between the superior temporal gyrus and the reward system of musicians when musicians listen to music with familiar and unfamiliar styles. **(A)** Music with Chinese style: Chinese-trained vs. Western-trained musicians. Stronger connections concluded edges among the right superior temporal gyrus, right midbrain, left amygdala, and left medial prefrontal cortex. **(B)** Music with Western style: Western-trained vs. Chinese-trained musicians. Stronger connections concluded edges among the left superior temporal gyrus, left midbrain, right amygdala, right medial prefrontal cortex, left dorsal striatum and right orbitofrontal cortex. The red lines represent the enhanced connections. The statistical results are presented in Tables 3, 4.

To study the relationship between the auditory system and reward system, we defined 6-mm radius spherical ROIs conclude left and right STG, ventral and dorsal striatum, medial prefrontal cortex, midbrain, amygdala, hippocampus, and orbitofrontal cortex in the functional connectivity analysis (Schott et al., 2007; Furukawa et al., 2014; Kruse et al., 2018; Zou et al., 2018). Then, we extracted the signals of ROIs during the task section and calculated the functional connections by using code, to further investigate four conditions (Chinese-trained musicians and Western-trained musicians listened to music with Chinese or Western style). In addition, we compared the connections between the conditions (Chinese music style: Chinese-trained musicians vs. Western-trained musicians and Western music style: Western-trained musicians vs. Chinese-trained musicians, Figure 4).

## Results

We find that there is no significant difference in terms of starting age, training years, weekly training hours, and total training hours (weekly training hours × the number of weeks in a year × training years) (these results are shown in Figures 1A–D). However, musicians have a significant difference in their familiarity with Chinese and Western music styles. Specifically, Chinese-trained musicians are more familiar with Chinese music style than Western-trained musicians. In addition, Western-trained musicians are more familiar with Western music style than Chinese-trained musicians (Figures 1E,F).

The activation of the left and right superior temporal gyrus of Chinese-trained musicians is stronger than that of non-musicians and the activation of the left superior temporal gyrus of Western-trained musicians is stronger than that of non-musicians during this experimental task. The three comparisons, which were Chinese-trained musicians vs. non-musicians under the Chinese music style condition, Chinese-trained musicians vs. non-musicians under the Western music style condition and Western-trained musicians vs. non-musicians under the Chinese music style condition passed FDR-correction, but the comparison of Western-trained musicians vs. non-musicians under the Western music style condition did not survive the correction (Figure 3 and Table 2). By comparing the connections between the superior temporal gyrus and the reward system of Chinese and Western-trained musicians, we find that the connection between the right superior temporal gyrus and reward system when Chinese-trained musicians listen to music with Chinese style is stronger than that of Western-trained musicians listening to it. For Western-trained musicians, the connection between the left superior temporal gyrus and reward system when they listen to music with Western style is stronger compared with Chinese-trained musicians (Figure 4 and Table 3, Table 4).

**TABLE 2:** Stereotaxic locations of significant clusters of activation. Stereotaxic locations were used to locate significant clusters of activation (Talairach and Pa, 1988).

**Chinese-trained musicians listening to music with Western style**						
**Region (AAL)**	**BA**	**MNI coordinates (mm)**			**T score**	**Cluster (voxels)**
		***x***	***y***	***z***		
Temporal_Sup_L	22	−63	−39	15	5.2762	54
Temporal_Sup_R	42	66	−18	9	5.4844	67

*L, left; R, right; AAL, anatomical automatic labeling; BA, Brodmann area; FDR-corrected *p* < 0.05; *p* = 0.00078572; and cluster size ≥621 mm^3^.*

**Table:** 

**Chinese-trained musicians listening to music with Chinese style**						
**Region (AAL)**	**BA**	**MNI coordinates (mm)**			**T score**	**Cluster (voxels)**
		***x***	***y***	***z***		
Temporal_Sup_L	22	−60	−39	15	4.2404	46
Temporal_Sup_R	42	63	−18	9	5.6102	85

*L, left; R, right; AAL, anatomical automatic labeling; BA, Brodmann area; FDR-corrected *p* < 0.05; *p* = 0.00086851; and cluster size ≥621 mm^3^.*

**Table:** 

**Western-trained musicians listening to music with Chinese style**						
**Region (AAL)**	**BA**	**MNI coordinates (mm)**			**T score**	**Cluster (voxels)**
		***x***	***y***	***z***		
Temporal_Sup_L	22	−66	−42	12	4.9514	91

*L, left; R, right; AAL, anatomical automatic labeling; BA, Brodmann area; FDR-corrected *p* < 0.05; *p* = 0.0006865; and cluster size ≥621 mm^3^.*

**Table:** 

**Western-trained musicians listening to music with Western style**						
**Region (AAL)**	**BA**	**MNI coordinates (mm)**			**T score**	**Cluster (voxels)**
		***x***	***y***	***z***		
Temporal_Sup_L	22	−66	−42	12	4.4412	74

*L, left; R, right; AAL, anatomical automatic labeling; BA, Brodmann area; FDR-uncorrected *p* < 0.05; *p* = 0.005; and cluster size ≥621 mm^3^.*

**TABLE 3:** The connection difference between the superior temporal gyrus and the reward system of different musicians when listening to music with Chinese style.

**Connection degree**	***t***	***p***
Superior temporal gyrus_R – Midbrain_R	2.57	0.015
Superior temporal gyrus_R – Amygdala_L	2.96	0.006
Medial prefrontal cortex_L – Midbrain_R	2.28	0.029
Medial prefrontal cortex_L – Amygdala_L	2.19	0.036

*Chinese-trained musicians vs. Western-trained musicians.*

**TABLE 4:** The connection difference between the superior temporal gyrus and the reward system of different musicians when listening to music with Western style.

**Connection degree**	***t***	***p***
Superior temporal gyrus_L – Amygdala_R	2.15	0.039
Amygdala_R – Midbrain_L	2.37	0.024
Midbrain_L – Orbitofrontal cortex_R	1.61	0.116
Dorsal striatum_L – Superior temporal gyrus_L	2.31	0.027
Orbitofrontal cortex_R – Medial prefrontal cortex_R	2.85	0.008
Medial prefrontal cortex_R – Dorsal striatum_L	2.13	0.041

*Western-trained musicians vs. Chinese-trained musicians.*

## Discussion

The familiarity of music styles caused by instrument training is an important factor in music recognition. We examined three groups of subjects, namely, Western-trained musicians, Chinese-trained musicians and non-musicians, to study brain plasticity under the influence of musical stimuli. Previous studies have found that different abilities in musical processing, such as reading musical scores (Bouhali et al., 2020), recognition of timbre (Mazzucchi et al., 1982), speech in-noise processing (Strait et al., 2012), and perception of pitch and rhythm (Liégeois-Chauvel et al., 1998; Matsui et al., 2013; Yu et al., 2017), are associated with the temporal lobe. Thus, the temporal lobe plays an important role in the perception of music (Satoh et al., 2001, 2003) and is involved in processing musical features (Patterson et al., 2002; Grahn and Rowe, 2009; Samson et al., 2011).

In this study, greater activation of the left superior temporal gyrus was found during Western-trained musicians listening to musical stimuli compared with non-musicians. Whereas Chinese-trained musicians had greater activation in the bilateral superior temporal gyrus than non-musicians. These results indicate that different music styles which musicians are exposed to during their daily training will affect their strategies of listening to music. Specifically speaking, exposure to Western music style due to long-term musical training will lead Western-trained musicians rely more on the processing of rhythmic features when they listen to music, which is reflected in the activation of the left STG. Rhythm recognition relies on temporal prediction in temporal processing. There is evidence that the left auditory cortex is mainly responsible for temporal processing (LaCroix et al., 2015) and that it has a higher degree of temporal sensitivity (Zatorre and Belin, 2001; Zatorre et al., 2002). Additionally, there is greater left lateralization in the brain activity of musicians compared to non-musicians during rhythm perception, particularly within the superior temporal gyrus (Limb et al., 2006). In some ways, regular rhythm and stress are considered fundamental features of Western music style, which provides evidence for the above statement (Levitin et al., 2012; London, 2012; Stevens, 2012; Ding et al., 2017). For Chinese-trained musicians, exposure to Chinese music style due to long-term musical training will lead them to rely more on the processing of melodic features when they listen to music, which is reflected in the bilateral STG activation. Rogalsky et al. have found that listening to the melody would lead to the activation of the bilateral STG (Rogalsky et al., 2011). Study on patients with the bilateral temporal lobe lesions also reported that these patients could not perceive melody (Peretz et al., 1994). Other studies found that activation of bilateral temporal lobes is correlated with familiarity with melodies (Groussard et al., 2010; Agustus et al., 2018). Additionally, researchers have found melody processing with more activity in the right auditory system (Peretz et al., 1994; Zatorre et al., 2002; Harms et al., 2014), which may be due to it is mainly involved in spectral processing and has higher spectral sensitivity (Zatorre and Belin, 2001; Zatorre et al., 2002; LaCroix et al., 2015). Part of Chinese traditional music has irregular beat attributes and melody is a prominent feature, which provides evidence for the above statement (Jiang, 1991; Stevens, 2012; Chen, 2016).

The results also show the differences in the functional connections between the superior temporal gyrus and the reward system when Chinese and Western-trained musicians listen to musical stimuli. These connections are stronger when musicians listen to the musical pieces from their familiar musical style as compared to listening to music from unfamiliar style. Studies have shown that implicit expectations of music are based on the history of listening to music and the implicit understanding of music rules. For example, the rhythm is considered highly regular and predictable and has been thought to contribute to our enjoyment of music (Levitin et al., 2012). Whereas the prediction of melody is an important aspect of music listening (Thiessen and Saffran, 2009; Egermann et al., 2013; Lappe et al., 2013). Meanwhile, exposure effects support the contention that accurate prediction is rewarding, familiar music will make musicians more enjoyable, which has been confirmed its presence for melodies (Wilson, 1979; Zald and Zatorre, 2011). Hence, just like prior exposure to a piece of music makes it more predictable. Musical training contributes to predicting upcoming musical events by raising the accuracy of the discrimination of musical material (Lappe et al., 2013). Familiarity with the music style contribute to musical pleasure, rhythm characteristics in Western music style as well as melody characteristics in Chinese music style may play an important role in this process.

There is evidence supporting a deep understanding and clear familiarity with the rules of musical structure have contributed to the anticipation of upcoming musical events, listeners will pay attention to whether it meets their expectations, in turn leading to emotional arousal (Salimpoor et al., 2011). The activity in the STG is correlated with the reward system when subjects listen to personally pleasurable music (Martínez-Molina et al., 2016; Gold et al., 2019). Therefore, although we do not obtain musicians’ rating of pleasure from the musical pieces, but we suggest that when the implicit expectation is not met (i.e., when Western-trained musicians listen to Chinese music or Chinese-trained musicians listen to Western music), the connection between the STG and reward system is low because the musical characteristics in the music style do not meet their predictions. This outcome is possibly caused by music that violates expectations at the beginning and will be considered unattractive because it prevents accurate predictions (Zald and Zatorre, 2011). These findings support that when musicians listen to music in their field of expertise, their clear familiarity and deep understanding of the musical characteristics in the musical style will lead to enhanced connectivity between the STG and reward system.

In general, we think that musicians will have a deeper understanding of the music style that they often hear during daily training, and familiar musical style can help musicians predict upcoming musical events. Acquired musical expertise of musicians can raise the prediction accuracy, which makes upcoming musical events meet their expectations. This process leads to musicians’ enjoyment of music, which is related to the increased connection between the STG and reward system. Furthermore, this result is consistent with our background information results. That is, Chinese-trained musicians are familiar with Chinese music style, which means they are more familiar with the rules and features of Chinese music than Western-trained musicians. The same holds for Western-trained musicians (Figures 1E,F). Considering that the musicians recruited in this study are typical Chinese and Western instrumentalists and that the experimental stimuli also contain typical cultural characteristics of Chinese and Western music styles, we think that musicians’ familiarity with culture attribute is also one of the reasons that leads to the differences in functional connectivity when they listen to Chinese and Western music. This helps explain the differences of the connections between the STG and the reward system of Chinese and Western-trained musicians when listening to music with Chinese or Western styles.

The results also show the laterality of the connections between the STG and the reward system. The connections of Chinese-trained musicians are partial to the right STG, and the connections of Western-trained musicians are partial to the left STG. Comparatively speaking, Western music style emphasizes rhythm, while Chinese music style emphasizes melody. The left hemisphere of the brain is mainly involved in temporal processing and rhythm recognition whereas the right hemisphere principally participates in spectral processing and melody recognition. These results explain the laterality of the circuit between the STG and the reward system in Chinese-trained and Western-trained musicians. In our results, there is no difference in connections of the STG and the reward system between musicians and non-musicians. We suggest that this finding occurred because non-musicians have less understanding of the rules of Chinese or Western music than experienced musicians. Thus, they hardly have implicit expectations based on the musical features in music style and just enjoy the music; therefore, they can always produce a reward response.

## Conclusion

This investigation researched trained musicians’ brain activities under condition of listening to music of the style in which they are experts or not experts. The results showed that Chinese-trained musicians activated the bilateral superior temporal gyrus and Western-trained musicians activated the left superior temporal gyrus during the task. When listening to the music in which they are experts, Chinese-trained musicians showed enhanced connections between the right superior temporal gyrus and the reward system compared with Western-trained musicians. However, Western-trained musicians showed enhanced connections between the left superior temporal gyrus and the reward system. This outcome indicates that the circuit that connects the superior temporal gyrus and reward system has different patterns between Chinese-trained musicians and Western-trained musicians when they are listening to music in which they were experts or not experts. The activities and connections in the task are related to the characteristics of the music style that musicians are exposed to in their daily training. Chinese-trained musicians and Western-trained musicians have laterality in processing the characteristics of music style they expertise. One of the reasons for the difference may also be the familiarity with culture attribute.

## Limitations

This study has some limitations. Although we mentioned that the cultural attributes of music style may lead to differences between Chinese and Western musicians in listening to familiar music style, the influence of musical instruments needs to be considered in the further exploration. Research on the cultural attributes suggests that musical instruments are potent cultural phenomena. They are embodiments of culturally based belief and value systems (Dawe, 2011). In this work, the instruments (guqin, erhu, and piano) trained by musicians are considered as typically traditional Chinese and Western instruments, and which are culturally specific. In addition, these musicians have been selected and tested. Thus, we believe that they are almost fully influenced by musical culture and can be the professional model for this study. Professional scales for familiarity of musical culture are necessary for us to distinguish the influence of musical instruments and culture. However, at present, there is no authoritative scale to measure the familiarity of musical culture. When the authoritative scale is developed, it will be helpful for enriching this hypothesis. Furthermore, the comparison of Western-trained musicians vs. non-musicians under the Western music style condition did not survive the correction, even though it showed a tendency similar to that in the Chinese music style condition. This may be due to the small sample size caused by the recruitment of subjects (we try our best to recruit the professional guqin and erhu players from conservatory of music, but guqin and erhu, the typically traditional Chinese instruments, are learnt by a small crowd). Thus, sample sizes should be increased in future studies.

## Data Availability Statement

The raw data supporting the conclusions of this article will be made available by the authors, without undue reservation.

## Ethics Statement

The study was done with the approval of the Ethics Committee of the School of Life Sciences and Technology at the University of Electronic Science and Technology of China. All procedures were carried out in accordance with approved guidelines. The patients/participants provided their written informed consent to participate in this study.

## Author Contributions

JL, DY, and PL contributed for ideas and design of experiment. SG, RD, JZ, and YL contributed for literature research and review. KL contributed for providing experimental sites and equipment. KP and PL contributed for selecting experimental stimuli. KP, JZ, YL, and YH contributed for recruiting subjects and collecting experimental data. SG, RD, YH, and YhL contributed for processing data. SG, RD, and JL contributed for making charts and writing manuscript. JL, CL, and DY contributed for reviewing manuscript and providing suggestions. All authors contributed to the article and approved the submitted version.

## Conflict of Interest

The authors declare that the research was conducted in the absence of any commercial or financial relationships that could be construed as a potential conflict of interest.

## Footnotes

1 http://cn.imslp.org/

2 http://music.163.com/

3 http://www.fil.ion.ucl.ac.uk/spm
